# The effect of vibration generated by demolition work on irradiation position accuracy of stereotactic radiotherapy system

**DOI:** 10.1002/acm2.13659

**Published:** 2022-05-29

**Authors:** Kaname Tanaka, Junji Suzuki

**Affiliations:** ^1^ Department of Radiation Therapy Toyota Memorial Hospital Toyota Japan

**Keywords:** CyberKnife, radiation therapy, stereotactic radiotherapy, vibration, vibration criteria

## Abstract

**Purpose:**

One existing building, adjacent to the radiotherapy building, where stereotactic radiation was done, was to be demolished to give space for the construction of a new building. However, we were concerned that the vibrations generated by this demolition work, which occurred within 2 m from the radiotherapy building, would affect the radiation position accuracy of the radiotherapy machine.

**Methods:**

To determine whether radiotherapy could be performed safely during the demolition period, we performed simulation tests involving the vibrations generated during demolition, measured these vibrations, and verified their effect on the irradiation position accuracy of the stereotactic radiotherapy system. For effective evaluations, tests were conducted assuming the maximum vibrations that could occur during actual demolition work.

**Results:**

The maximum displacement of the vibrations generated by the simulated demolition work was 3.30 µm on the floor of the treatment room and 4.68 µm at the ceiling.

**Conclusions:**

The results of the vibration measurements exceeded the limits of the criteria applicable to the electron beam system. However, the accuracy of the irradiation position of the stereotactic radiotherapy system remained unchanged during these vibrations. Therefore, the vibrations had no impact on radiotherapy safety, and radiotherapy was continued during the demolition work while coordinating with the demolition workers as necessary.

## 1 INTRODUCTION

The main hospital building adjacent to our facility, where stereotactic radiotherapy was performed, was planned for demolition to make way for reconstruction. Although the radiotherapy building was not included in the demolition plan, there were concerns that the vibrations generated from the demolition work would affect the accuracy of the irradiation position of the radiotherapy machine, because the demolition site was only 2 m away from the radiotherapy building. Therefore, we aimed to assess the intensity of the vibrations that could affect the radiotherapy machine and verify the safety in continuing the radiotherapy treatment.

Thus far, only few studies have described the effects of vibrations on radiotherapy; however, clear regulations for controlling the vibrations in the stereotactic radiotherapy treatment room remain unavailable. We also inquired about machine specifications to the stereotactic radiotherapy system vendor, who informed us that there are no existing specific vibration requirements. Hindmarsh et al. quantitatively evaluated the effects of vibrations generated by construction work on daily radiotherapy^1^; however, the effect of vibrations from demolition work was not highlighted. They reported that the propagation of vibrations from construction is complicated, and estimating their effects accurately is difficult. In addition, they cited the installation criteria for the general linear accelerators (LINACs) as an indicator of vibrations and mentioned the vibration criteria (VC) proposed by Gordon.^2^ Gordon^2^ previously provided a useful table for the maximum root mean square (RMS) particle velocity limits based on VC curves.

VC can be divided into several categories: vibrations that cannot be felt can be subdivided into categories A, B, C, D, and E with RMS particle velocity limits of 50, 25, 12.5, 6, and 3 µm/s, respectively. The smaller the velocity limits, the more severe the restriction; therefore, VC‐E entails the most stringent criteria among categories from A to E. However, VC‐D involves a maximum RMS particle velocity of 6 µm/s and is applied for electron microscopy and electron beam systems. In general, radiation therapy machines generate photons by accelerating electron beams; therefore, the VC‐D criteria could be a useful reference for this study.

Ungar et al.^3^ outlined the VC for health‐care facility floors and presented a summary of the criteria provided by MRI equipment suppliers, which were equivalent to or stricter than general hospital constraints. However, they failed to consider radiotherapy machines.

The operational status and location of radiotherapy machines vary depending on the facility; therefore, it is difficult to determine the safe performance of radiotherapy during demolition solely based on information from the abovementioned literature. As reported by Hindmarsh, it is not feasible to investigate the effects of vibrations once the demolition work commences because it may lead to the discontinuation of the acceptance of radiotherapy patients. Therefore, in this study, a demolition–simulation experiment was conducted to determine whether radiotherapy can be performed safely during the demolition period. In this experiment, vibrations similar to those generated during demolition work were generated, the generated vibrations were measured, and their effects on the radiotherapy machine were evaluated. At our facility, two radiotherapy machines are currently in operation. One is a general LINAC, and the other is a stereotactic radiotherapy machine. In this study, we verify the effects of vibrations on the stereotactic radiotherapy machine that is closer to the demolition work site and requires considerably accurate treatment.^4^

Specifically, we conducted an experiment simulating demolition work that generated vibrations, measured these generated vibrations, verified their effects on the accuracy of the irradiation position of the stereotactic radiotherapy system, and evaluated whether radiotherapy could be performed safely.

## 2 METHODS

### 2.1 Generation and measurement of vibrations

For a safer evaluation, the tests were conducted during weekend assuming the maximum vibrations that could be generated during the actual demolition work. For the tests, we employed equipment that would be utilized during the actual demolition work, and a part of the actual demolition work was completed.

The equipment used to generate vibrations was ZAXIS 210K (Hitachi Construction Machinery Co., Ltd., Tokyo, Japan). This heavy machine can be used to perform various demolition tasks by simply changing the attachments. Two types of attachments were used to generate the vibrations: a hydraulic breaker, which demolished objects by striking them with a chisel, and a plier‐shaped crusher. Table 1 lists the contents of the demolition work simulation tests: heavy machine running test, heavy machine sudden start and stop test, retaining wall demolition test using a hydraulic breaker, and retaining wall demolition test using a crusher. The first two tests were conducted at the demolition work site, whereas the latter two tests targeted the retaining wall at the boundary between the demolition work area and parking area.

**TABLE 1:** Contents of the demolition work simulation tests (a heavy machine running test, a heavy machine sudden start and stop test, a retaining wall demolish test using a hydraulic breaker, and a retaining wall demolish test using a crusher)

Examination	Contents of examination
Running	Heavy machine with steel caterpillar move forward and back
Sudden start and stop	Heavy machine with steel caterpillar suddenly start and stop
Breaking	Heavy machine break retaining walls
Crushing	Heavy machine crush retaining walls

*Note*: The former two tests (a heavy machine running test and a heavy machine sudden start and stop test) are conducted in the demolition work area, and the latter two tests (a retaining wall demolish test using a hydraulic breaker and a retaining wall demolish test using a crusher) target the retaining wall at the boundary between the demolition work area and the parking area.

**FIGURE 1:**
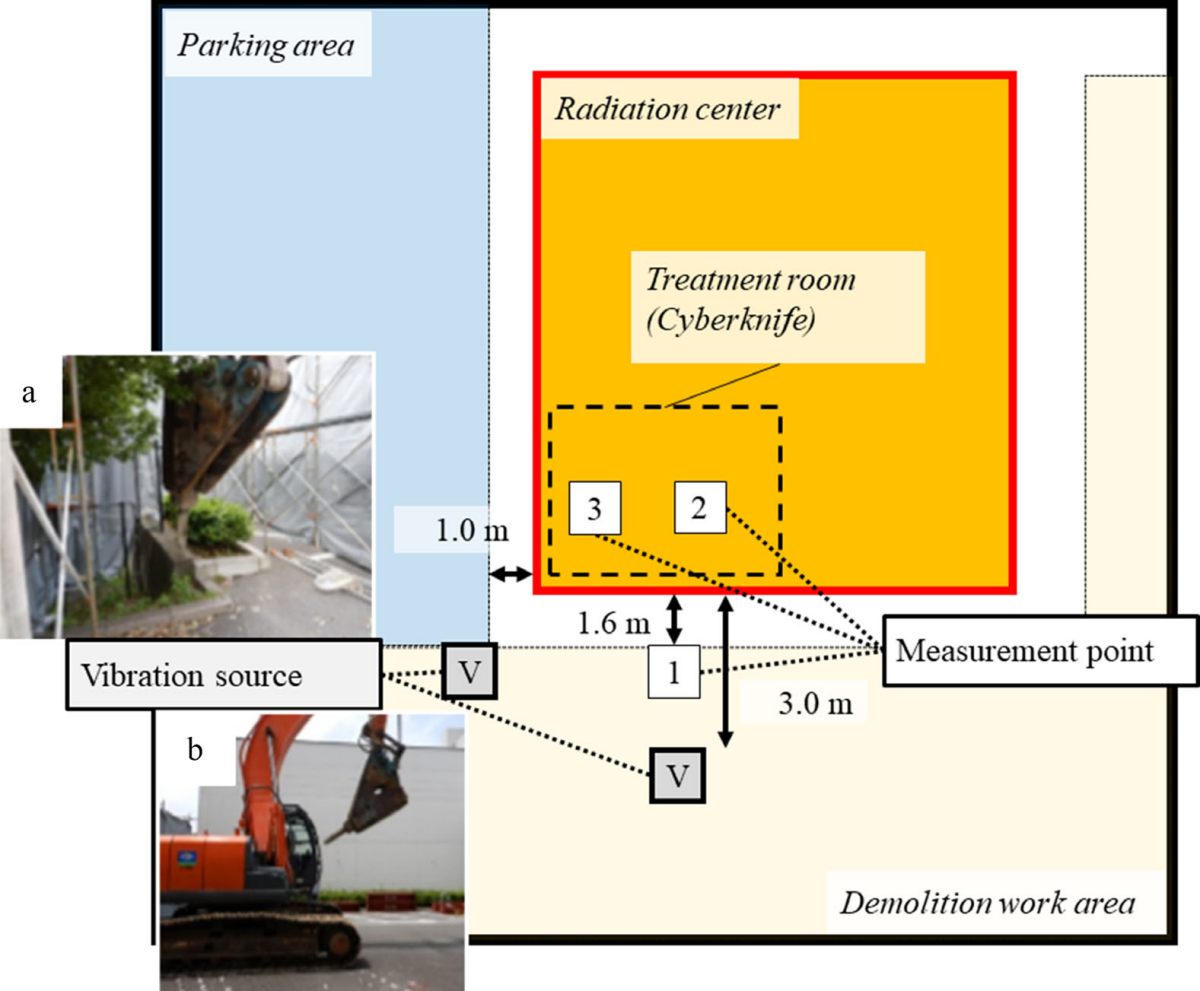
Location of measurement points and vibration sources. Measurement point 1 was located 1.6 m away from the outer wall of the radiotherapy building, measurement point 2 was located on the floor near the radiotherapy machine in the treatment room, and measurement point 3 was located at the ceiling of the treatment room. Image (a) shows “breaking” and “crushing.” Image (b) shows “running” and “sudden start and stop.”

The vibrations generated were measured using a vibration‐level meter. The positional relationships during the measurements are shown in Figure 1. Measurement point 1 was located 1.6 m away from the outer wall of the radiotherapy building, measurement point 2 was located on the floor near the radiotherapy machine in the treatment room, and measurement point 3 was located at the ceiling of the treatment room. A VM‐53A vibration‐level meter (Rion, Tokyo, Japan), DA20 data recorder (Rion, Tokyo, Japan), NITAN‐01 level waveform monitor (Nihon Industrial Technical Center, Tokyo, Japan), and a DS2100 waveform analyzer (Ono Sokki, Kanagawa, Japan) were used.

The evaluation scales for vibrations include the displacement amplitude (displacement), velocity amplitude (velocity), acceleration amplitude (acceleration), and vibration level.^5^ In this study, each scale was selected on the basis of its purpose. The vibration‐level waveforms were obtained in decibel units using a vibration‐level meter. The decibel (dB) is a relative unit of measurement equal to one‐tenth of that of a bel (B). It is defined by the following formula,^5^ when the acceleration is *A* and the standard vibration acceleration is *A*_0_.

$$\mathrm{Vibration}\;\mathrm{level}\;\left(\mathrm{dB}\right)\;=\;20\log_{10}\left(A/A_{0}\right)$$

In this manner, the vibration level can be converted into acceleration, and this acceleration (m/s^2^) can be expressed by the following formula, where *f* is the frequency (s^−1^), and *D* is the displacement (m):

$$\mathrm{Vibration}\;\mathrm{acceleration}\;\left(m/s^{2}\right)\;\;={\left(2\pi f\right)}^{2}\;D$$

The displacement, velocity, and acceleration were obtained from the vibration‐level waveform using a waveform analyzer at µm, µm/s, and µm/s^2^, respectively.

### 2.2 Measurement of irradiation position accuracy for stereotactic radiotherapy machine

To perform the aforementioned vibration measurements, the position accuracy of the treatment machine was verified using a method that does not generate radiation to prevent any interference with the vibration measurements conducted in the treatment room. At our facility, the CyberKnife M6 system (CK, Accuray Inc., Sunnyvale, CA, USA) was used as the stereotactic radiotherapy machine. CK consists of a LINAC mounted on a robotic manipulator; two X‐ray generators mounted on the treatment room ceiling for kV image acquisition; and two flat‐panel detectors located on the treatment room floor, enabling beam delivery from any point on a hemisphere around the patient and automated image‐guided corrections of motion by the robot, which reduces the exposure of normal tissues and organs from risk.^6^ The LINAC of the CK is equipped with a central axis laser.^7^ The center of the laser beam and the radiation beam are adjusted to match, within 1.0 mm.

**FIGURE 2:**
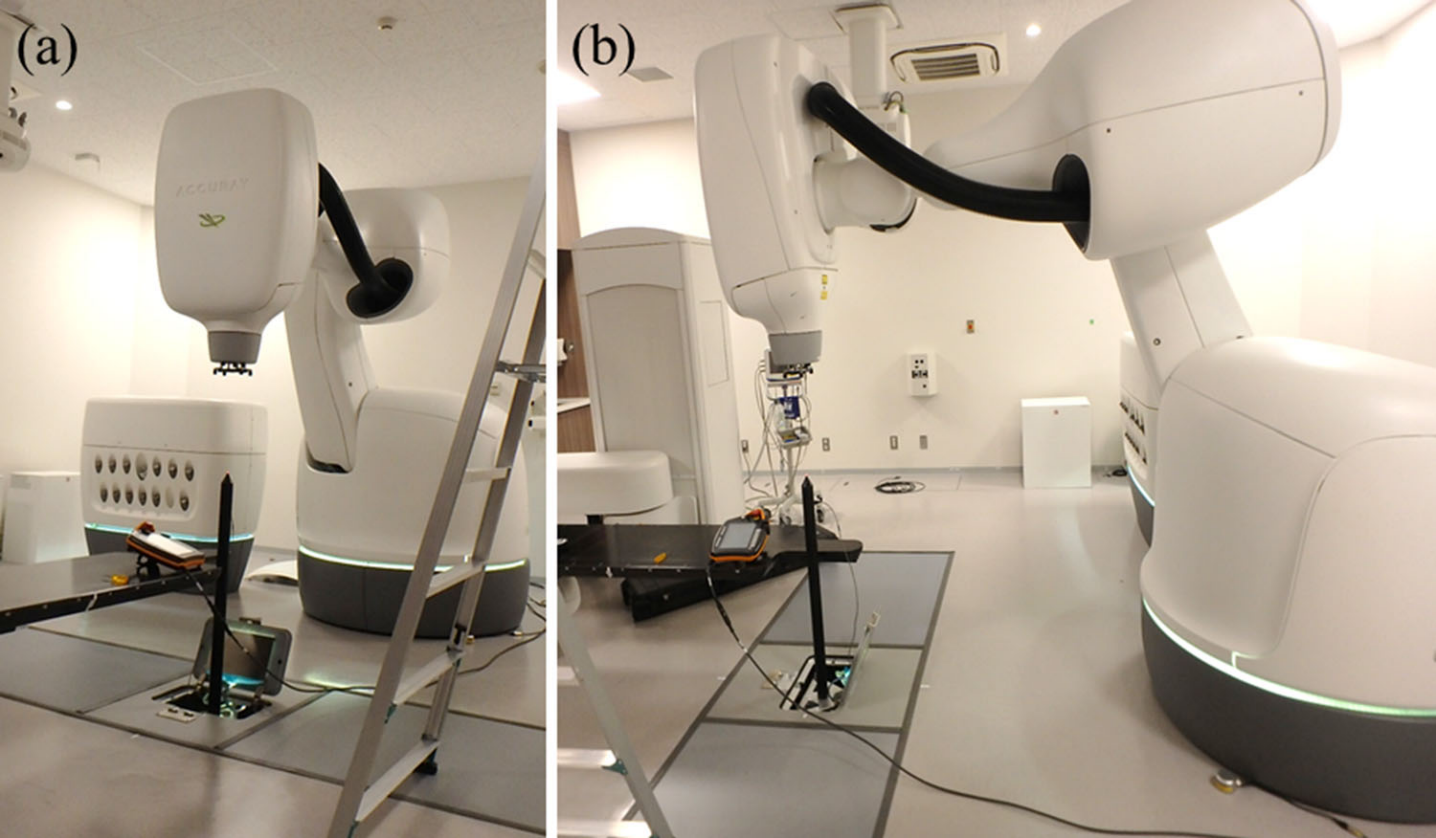
Front view (a) and side view (b) of the study setting. Isopost was installed in the treatment room; the laser beam was projected vertically

The laser beam was used as a substitute for the radiation beam. Simultaneously, we visually observed changes in the laser and experienced vibrations in the treatment room. As shown in Figure 2, a device that can quantitatively detect the laser beam (isopost, Accuray Inc., Sunnyvale, CA, USA) was installed in the treatment room, and the laser beam was projected vertically to generate vibrations and record the laser beam over time. This method can provide quantitative data on voltage values over time. Because it was impossible to generate vibrations continuously, it was necessary to employ a method to assess the effect of vibrations that vary overtime on the machine.

**FIGURE 3:**
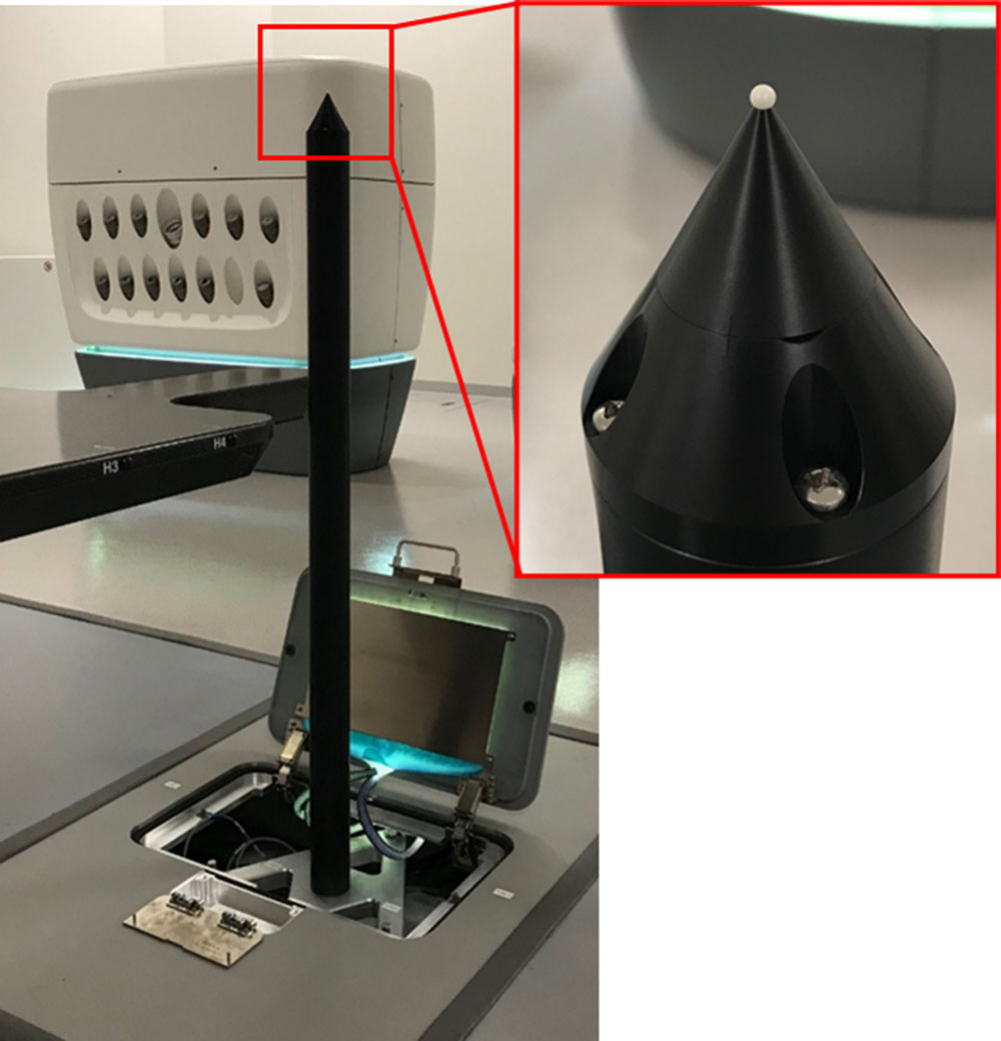
Overview of the isopost and isocrystal. The isopost is equipped with a spherical light‐sensitive detector with a diameter of approximately 1.5 mm (isocrystal) at the tip. When the isocrystal is irradiated with laser light, the amount of light is converted to a voltage value

As shown in Figure 3, the isopost is equipped with a spherical photoreceptor (isocrystal, Accuray Inc.) with a diameter of approximately 1.5 mm.

When an isocrystal is irradiated with laser light, the light intensity is converted to a voltage value. Thus, the measured laser light was recorded as the voltage value. The intensity of the laser light gradually decreases by approximately 0.3 V/h; this should be considered when evaluating the absolute value of the voltage value. To understand the error introduced by the measurement method better, several measurements were performed before the vibrations, and a standard deviation of approximately 6–10 mV was obtained.

## 3 RESULTS

### 3.1 Measurement results for vibrations at each measurement point

**FIGURE 4:**
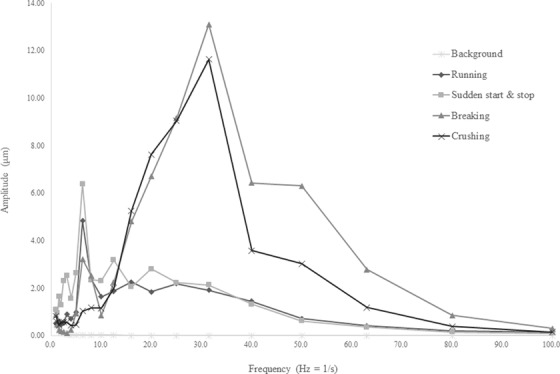
Zero‐peak displacement at measurement point 1 (demolition worksite). The displacement recorded during the retaining wall demolition tests at the demolition worksite is large, with a high frequency range of 30 Hz

**FIGURE 5:**
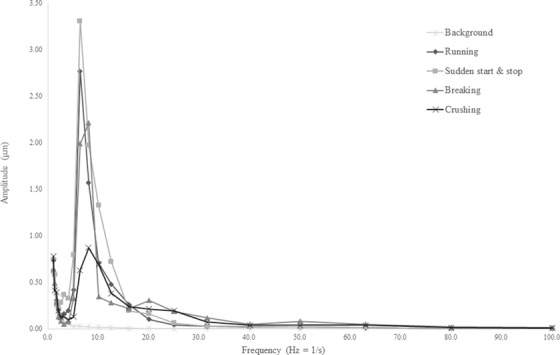
Zero‐peak displacement at measurement point 2 (treatment room floor). The displacement increased at a frequency of 3 Hz and beyond. The maximum displacement was 3.30 µm at a frequency of 6.3 Hz

**FIGURE 6:**
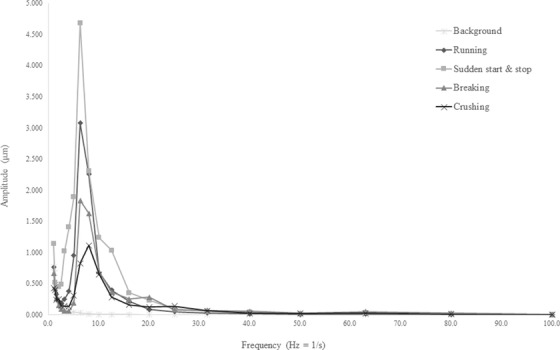
Zero‐peak displacement at measurement point 3 (treatment room ceiling). The displacement increased at a frequency of approximately 3 Hz and beyond. The maximum displacement was 4.68 µm at a frequency of 6.3 Hz. Although the results of the treatment room floor and ceiling have similar frequency characteristics, the displacement at the treatment room ceiling is larger than that at the treatment room floor

Figures 4—6 show the vibration displacements at each frequency converted from the vibration levels recorded at each measurement point. For all the measurement points and tests, higher vibration displacements were recorded compared with those under the untested condition. At measurement point 1, which was located at the demolition work site and the closest to the vibration source, a higher vibration displacement was recorded compared with those at the other measurement points. Figure 5 shows that the vibrations generated during the tests involving the operation of heavy machinery in the treatment room were stronger than those in the retaining wall demolition test. The displacement measured at the demolition work site exhibited a larger peak than that in the treatment room.

The largest displacement was observed in the retaining wall demolition test conducted at the demolition work site using the hydraulic breaker. However, the effect of the vibrations generated during this test was relatively weak in the treatment room. Furthermore, the displacement recorded during the retaining wall demolition tests at the demolition work site was large with a high frequency of 30 Hz. In both tests, the displacements recorded on the floor of the treatment room began to increase when the frequency exceeded 3 Hz. Comparing the results of each test on the treatment room floor, the largest vibration occurred during the heavy machinery rapid start/stop test, with a displacement of 3.30 µm at a frequency of 6.3 Hz. Similar results were obtained at the ceiling of the treatment room, with a displacement of 4.68 µm at the same frequency of 6.3 Hz. Although the vibrations at the treatment room floor and ceiling had similar frequency characteristics, the displacement at the treatment room ceiling was greater than that at the treatment room floor. Moreover, the vibrations caused by the movement of the heavy machine exhibited a frequency of 6.3 Hz at all the measurement points. To evaluate the RMS particle velocity, the vibration‐level waveform measured at each measurement point was converted into velocity amplitudes using a waveform analyzer. The maximum velocity amplitude was 2592 µm/s with a frequency of 31.5 Hz, which was obtained at measurement point 1. Amplitudes of 131 and 185 µm/s for a frequency of 6.3 Hz were noted at measurement points 2 and 3, respectively. At measurement points 2 and 3, located on the treatment room floor and ceiling, respectively, the amplitudes exceeded 6 µm/s, which is the limit of the VC‐D category.

### 3.2 Measurement results for irradiation position accuracy in each test

**FIGURE 7:**
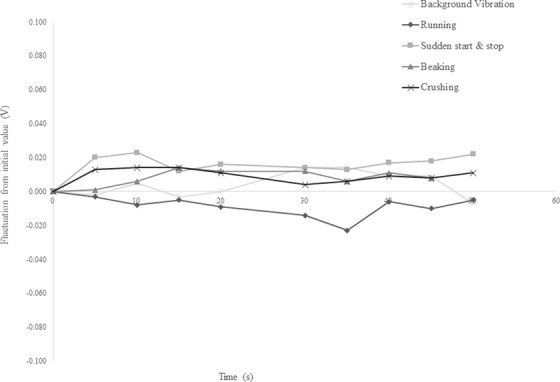
Fluctuation from initial value in each examination. The figure shows the change in the voltage values acquired by the isocrystal in each test from the initial value, at intervals of 5 s. The absolute value of the change in all the tests exceeded the background value

Figure 7 shows the change in the voltage values acquired by the isocrystal in each test from the initial value at intervals of 5 s. The absolute value of the change in all tests was slightly larger than the background value. Table 2 presents the average values and standard deviations of the changes observed in each test. In all the tests, no results exceeded the error introduced by the measurement method. Furthermore, no visible changes in laser light were observed, and no vibrations were observed in the treatment room.

**TABLE 2:** Mean and standard deviation of voltage of isocrystal in each examination

	Voltage of isocrystal (V)				
	Background vibration	Running	Sudden start and sudden stop	Breaking	Crushing
Mean (SD)	0.004 (0.007)	−0.008 (0.006)	0.015 (0.007)	0.007 (0.005)	0.009 (0.005)

*Note*: This measurement method had a standard deviation of approximately 6–10 mV. No results were found that exceeded the error caused by the measurement method.

## 4 DISCUSSION

Based on the vibration measurements, the test vibrations were found to propagate the treatment room.

The maximum vibration displacement at the treatment room floor was 3.3 µm, which is larger than the value of 2.35 µm reported by Hindmarsh et al.^1^ the vibration displacement at the treatment room ceiling was even greater, at 4.67 µm.

In a study by Hindmarsh, the resonant frequency was cited as the cause of the increase in the vibration amplitude, suggesting that it depends on the material of the radiotherapy machine, couch, and treatment room, as well as the gantry angle. In this study, as shown in Figures 4, 5, 6 , the vibrations generated in the same test were measured as close frequencies even when the measurement points differed. However, as noted in Hindmarsh's study, vibrations are transmitted through the ground to the building and are affected by vibrations within the building; hence, accurate predictions are not always possible. The most notable finding was that the maximum RMS particle velocity limit of the VC‐D category exceeded at measurement point 2. Exceeding this limit does not directly imply that the treatment needs to be discontinued; however, it is an important factor in making this decision.

In addition, the results obtained for the treatment room ceiling would not be significant if a general LINAC was considered, as in Hindmarsh's work. However, a CK system, which includes X‐ray generators for position‐matching imaging at the ceiling, was considered in this study. Hence, the results for the treatment room ceiling exceeded the limit of the VC‐D category. These findings suggest a need for vibration testing. Although position‐matching imaging is important, the imaging time is as short as 100 ms. Hence, it is unlikely to constitute a major problem during the occurrence of vibrations. In addition, position‐matching imaging is performed more than a dozen times per fraction, and the number of imaging sessions is even greater across the entire treatment. Hence, even if the result of position matching at one instance includes errors owing to sudden vibrations, the effect on the entire treatment is considered negligible. Therefore, this study focused on the accuracy of the irradiation position of the radiation beam rather than position‐matching imaging; however, the effect of vibrations on the position‐matching system warrants further research. A related description is provided in TG135.^7^ In addition, it is necessary to check whether the geometric arrangement has changed before and after the occurrence of vibrations.

As shown in Table 2, the standard deviation for each vibration test was within the measurement error. The vibrations measured in the treatment room reached the lowest vibration levels experienced by humans^8^; however, the time beyond that level was short (approximately 0.2 s), and we were unable to feel these vibrations. The generated vibrations were considered to have propagated along the floor of the treatment room based on the results of the vibration measurements. Nevertheless, because these vibrations were excessively small, their influence on the irradiation position accuracy could not be detected using this test method.

Therefore, it is important to establish a measurement method to verify the position accuracy of the treatment machine.

In a study by Hindmarsh, weekly evaluations of the LINAC isocenter and laser alignment were performed using a simplified Winston–Lutz test, which is a popular and well‐known test to verify whether the radiation isocenter coincides with the mechanical isocenter in a LINAC system.^9^, ^10^, ^11^ However, this method cannot be applied to CK, because CK is a machine that can emit radiation from multiple directions in three dimensions. Because isolating the cause can be complicated, a single vertical beam must be measured. As the magnitude of vibrations fluctuates, it is necessary to employ a method that enables the continuous collection of quantitative data. One such measurement method is automatic quality assurance, which is a popular and simple test to verify the irradiation position accuracy of CK,^7^ such as the Winston–Lutz test. In this method, the gantry moves to the irradiation position to perform irradiation. Therefore, the timing of large vibrations may overlap with the timing of this movement to the irradiation position. Despite the existence of several other quality assurance methods for CK, few can be used to solve the aforementioned problems. Although this study focused on the irradiation position accuracy of CK, the measurement method should be carefully selected while considering the effect of the position‐matching process and irradiation dose. Considering that the purpose is to determine whether radiotherapy is possible, large vibrations need not be emphasized, and a measurement method that assumes small vibrations that are difficult to determine is recommended. This method should serve as a simple geometric system, and a more rigorous method is desirable to ensure safety. The method adopted in this study is effective; however, further improvements are possible.

Based on the abovementioned findings, discussions were held with radiotherapy staff, medical physicists, treatment machine vendors, and demolition crews. Although the limit values of the VC‐D category, considered the initial indicator, were exceeded, it was agreed that this would not affect the machine overall. Ideally, vibrations exceeding those generated during demolition work tests should not occur; however, radiotherapy staff should be informed in advance in the event of such vibrations. Furthermore, accurately determining the effect of vibrations on radiotherapy is difficult; in actual clinical practice, it is also important to consider the benefits to patients and adjust the treatment schedule in collaboration with demolition crews.

## 5 CONCLUSIONS

To determine whether radiotherapy can be performed safely during demolition, simulation tests were conducted to generate vibrations, which were measured, and their effect on the irradiation position accuracy for the stereotactic radiotherapy machine was verified. Based on vibration measurements, the limit of the VC‐D category (the criterion applied to electron beam systems) was found to have been exceeded; however, the irradiation position accuracy remained unchanged owing to the vibrations of the stereotactic radiotherapy system. Therefore, the vibrations had no effect on the machine, and radiotherapy was continued during the demolition work period while coordinating with the demolition crews as needed.

## AUTHOR CONTRIBUTIONS

Kaname Tanaka contributed to the design and implementation of the research, to the analysis of the results, and to the writing of the manuscript.

Junji Suzuki contributed to the design and implementation of the research, to the analysis of the results, and supervised this study.

## Supporting information



Supporting Information

## Data Availability

The data supporting the findings of this study are available from the corresponding author upon reasonable request.
