# Supplementary material for: The effect of vibration generated by demolition work on irradiation position accuracy of stereotactic radiotherapy system
Source: J Appl Clin Med Phys. 2022 May 29;23(9):e13659. doi: 10.1002/acm2.13659 (PMC9512358; doi:10.1002/acm2.13659)
Supplement: Supplementary file 1 — Supporting Information [file ACM2-23-e13659-s001.docx]

**The effect of vibration generated by demolition work on irradiation position accuracy of stereotactic radiotherapy system**

Kaname Tanaka^1^, Junji Suzuki^1^

^1^ Department of Radiation Therapy, Toyota Memorial Hospital, Aichi, Japan

Tel: +81-0565-24-7306; Email: [kaname_sato@mail.toyota.co.jp](mailto:kaname_sato@mail.toyota.co.jp)

**Running title: Effect of vibration on radiotherapy**

**Author Contributions**

Kaname Tanaka contributed to the design and implementation of the research, to the analysis of the results and to the writing of the manuscript.

Junji Suzuki contributed to the design and implementation of the research, to the analysis of the results and supervised this study.

**Acknowledgements**

The authors are grateful to Dr Komori for his support in submitting this paper.

Mr. Yahagi and Mr. Ushimaru helped us to measure the machine.

Mr. Okada, Mr. Asakura, Mr. Maeda and Mr. Nakamura helped us to measure the vibration.

**Conflict of interest**

No conflicts of interest.
